# High-Cycle, Low-Cycle, Extremely Low-Cycle Fatigue and Monotonic Fracture Behaviors of Low-Carbon Steel and Its Welded Joint

**DOI:** 10.3390/ma12244111

**Published:** 2019-12-09

**Authors:** Younghune Kim, Woonbong Hwang

**Affiliations:** 1 Graduate Institute of Ferrous Technology, Pohang University of Science and Technology, 77 Cheongam-Ro, Nam-Gu, Pohang 37673, Korea; 2 Department of Mechanical Engineering, Pohang University of Science and Technology, 77 Cheongam-Ro, Nam-Gu, Pohang 37673, Korea

**Keywords:** low-carbon steel, fatigue modes, extremely low-cycle fatigue, fatigue test, fatigue transition

## Abstract

Low-carbon steels are commonly used in welded steel structures and are exposed to various fatigue conditions, depending on the application. We demonstrate that the various transitions in the fracture mode during fatigue testing can be distinguished by their different cyclic response curves and microstructural features after fracture. Fractography, surface damage micrographs, and microstructural evolution clearly indicated the transition of the fracture modes from high-cycle to low-cycle, extremely low-cycle fatigue, and monotonic behavior. The high-cycle fatigue mode showed initial cyclic softening, followed by cyclic stabilization, and showed inclusion-induced crack initiation at fish-eyes, while the low-cycle fatigue mode showed initial cyclic hardening followed by cyclic stabilization, where fractography images showed obvious striations. In addition, the extremely low-cycle fatigue mode showed no cyclic stabilization after initial cyclic hardening, which was characterized by quasi-cleavage fractures with a few micro-dimples and transgranular cracking, while the monotonic fracture mode predominantly showed micro-dimples.

## 1. Introduction

Low-carbon steels generally contain 0.05 to 0.2 wt.% (extensively up to 0.3 wt.%) carbon along with other alloying elements, such as manganese and silicon. Such steels are generally used for structural applications, where the strength and ductility can be optimized via thermo-mechanical controlled rolling (TMCR) or heat-treatment processes. For practical application, steel parts are usually joined by welding, which can introduce residual stresses and heterogeneous microstructures [1,2] that can result in locally inferior mechanical properties compared to the base material. For example, the fatigue life of welds is shorter than that of the base material due to welding defects [3,4] or heat-affected zone (HAZ) softening [5,6], where fatigue fracture occurs in these regions. Low-carbon steels are readily joined with several common fusion welding processes such as plasma arc welding (PAW) and gas tungsten arc welding (GTAW), but some studies [7,8,9] reported that friction stir welding process as an solid state welding showed superior fatigue strength due to the synergetic effect of microstructure, superior tensile properties and favorable residual stress, which inhibit the growth of cracks compared to other joints. Welded steel structures are subjected to cyclic loading conditions from high-cycle fatigue (HCF) to low-cycle fatigue (LCF), or even to extremely low-cycle fatigue (ELCF), whereas uniaxial loading results in monotonic fracture (MF).

**Figure 1:**
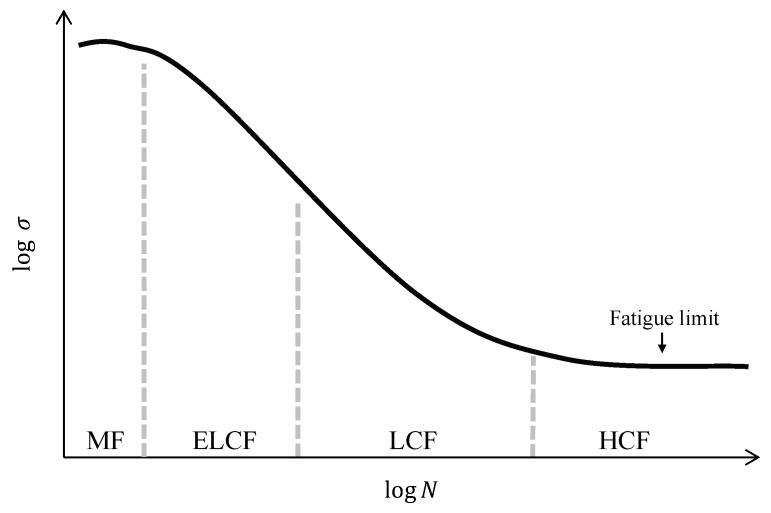
Schematics of classified fatigue modes [10]; abbreviations of fatigue modes are used as MF for monotonic fracture, ELCF for extremely low-cycle fatigue, LCF for low-cycle fatigue, and HCF for high-cycle fatigue.

The relevant fatigue fracture modes can be identified by analyzing the fatigue life and stress levels, as illustrated by Figure 1 [10]. 

The elastoplasticity theories are generally assumed on the basis of the decomposition of the total strain into elastic and plastic components so as to treat the elastic and plastic parts of the strains separately [11,12].

Previous studies focused on differentiating the classical HCF and LCF modes, and ELCF from LCF. The HCF mode is stress controlled and associated with relatively low stress levels that are relevant to predominantly elastic components [13]. In this mode, the fatigue life is >10^4^ cycles. In contrast, the LCF mode is strain controlled and related to large strain amplitudes or high stress levels that produce both plastic and elastic components at each cycle [14], where fatigue lives are <10^4^ cycles. The ELCF mode is between the MF and LCF modes and is controlled by the ductility and cyclic strain; initial damage under tensile conditions is followed by ductility exhaustion during cyclic loading until crack propagation occurs [15,16]. The fatigue life of ELCF is very short (<100 cycles). In other studies, specific fatigue modes and stress levels of stainless steels, copper alloys, aluminum alloys, and Ti alloys as well as low-carbon steels have been extensively investigated [14,15,16,17,18,19,20,21,22,23,24]. 

However, little attention has been paid to the consecutive fatigue transitions of low-carbon steel depending on cyclic response curves and fractography in addition to strain (stress) levels and fatigue life.

Moreover, for most structural applications, the different fatigue modes and fatigue lives of the base material and welded joints at various strain (stress) amplitudes have not been thoroughly elucidated, even though this is fundamental information for defining engineering safety factors.

The present study compared the fatigue behavior of a low-carbon steel with its welds using cyclic response curves and fractography over a range of strain amplitudes in order to describe the transitions between the HCF, LCF, ELCF, and MF modes, which is critical for designing durable structures using this material.

## 2. Materials and Methods 

### 2.1. Test Materials and Welding

The test material was a 4 mm-thick low-carbon steel sheet, which was a thermo-mechanical controlled rolled coiled tubing (CT) steel [4] with the chemical composition shown in Table 1.

Thermo-mechanical controlled rolling is a controlled process, extending from slab reheating, via rolling, up to and including (accelerated) cooling and, where necessary, tempering. Rolling is performed in accordance with a defined rolling-pass schedule, with finish rolling in the non-recrystallised austenite [25,26]. The essential effect of thermo-mechanical controlled rolling is the attainment of the fine grain, this being the necessary basis for the combination of high mechanical strength and toughness with only low alloying-element contents. For instance, Gladman [27] mentioned a precipitation strengthening up to 240 MPa containing 0.09 wt.% Nb on the assumption of complete NbC precipitation at the nanoscale. Funakawa et al. [28] reported the increased yielding strength up to 300 MPa due to the precipitation of nanometer size (Ti,Mo)C carbides. CT steel is one of the thermo-mechanical controlled steels used in oil field industries for applications such as work strings, drill, siphon, and velocity strings as well as sucker rod systems [29].

In the welding experiments, a plasma arc welding process was adopted to weld the low-carbon steel sheets. A single pass was conducted with a current of 250 A at a travel speed of 250 mm/min using a Powwel 500XP machine (Powwel Co., Ltd., Seoul, South Korea). An AWS A5.18:ER70S-6 filler wire was fed at a rate of 1500 mm/min, and 100% argon shielding gas was supplied at a flow rate of 1.5 L/min on the front surface and at 20 L/min to the back surface. After welding, a stress relief heat treatment (SRHT) was conducted at 600 °C for 15 min to relieve residual stresses [30].

### 2.2. Tensile and Fatigue Testing

**Figure 2:**
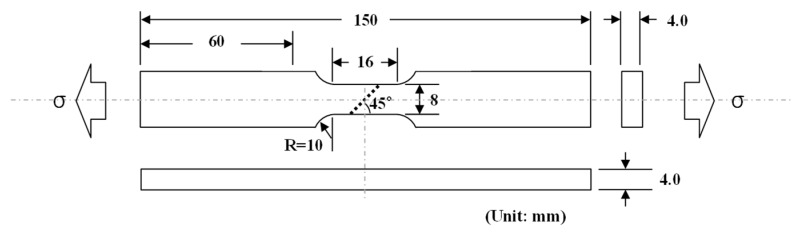
Axial fatigue specimen geometry; all units are in millimeters of tolerance within ± 0.05 mm, with the stippled line indicating the welding zone.

Tensile and fatigue tests of the base material (BM) and welded joints (WJ) specimens were performed using a 100 kN MTS closed-loop servo-hydraulic materials testing machine. The tensile and fatigue test specimens were machined from steel sheets cut longitudinally to the rolling direction. In the present study, the BM specimens were composed only of the base metal that retains the TMCR microstructure. The WJ specimen were composed of the weld metal where the portions of the base metal and filler metal has been completely melted during welding, the HAZ where mechanical properties or microstructure have been altered from the base metal by the heat of welding, and the base metal. The geometry of the fatigue specimens is shown in Figure 2. A 45° bias welding design was adopted [31], which allows a smoother transition between the mechanical properties of the base material and welds, while increasing the strength and decreasing the ductility by distributing the welding stresses over a larger area than a 90° bias. After machining, a smooth surface finish was obtained by polishing with emery papers of increasing fineness (320 down to 1000 grit). Tensile tests were conducted at a constant strain rate of 6.7 × 10^−3^ s^−1^. Fatigue tests were conducted in an identical manner for both the BM and WJ specimens at 0.05 Hz to 0.033 Hz under total strain control for total strains of ±0.5%, ±1.0%, ±1.5%, ±2.0%, ±2.5%, and ±3.0% using a triangular strain waveform with zero mean strain at a constant strain rate of 4 × 10^−3^ s^−1^. A 12-mm extensometer was used with the 16-mm-gauge-length specimens. Henceforth, the abbreviations BX and WX are used for the BM and WJ specimens, respectively, where X is the strain; for example, B0.5 is the BM specimen tested at 0.5% nominal strain. 

### 2.3. Microscopy

Before observing the microstructure, immersion etching was performed using a standard 2% nital solution (2% nitric acid in ethanol) followed by rinsing in ethanol and drying in hot air. Microstructural characterization was performed on mounted cross-sections of each sample type. Wet grinding was performed using SiC papers up to 2000 grit, followed by fine polishing using diamond and silica suspensions. Metallographic studies were performed using an Olympus AX70 (Olympus Corporation, Tokyo, Japan) inverted metallurgical microscope. The Future-Tech FM-700 Vickers hardness tester was applied with a load of 9.81 N and a dwell time of 10 s to the points, regularly 0.3 mm distanced from the center of the weld metal. In addition, high-magnification fractographs of ultrasonically cleaned samples were conducted using a JEOL JSM-6610LV scanning electron microscope (SEM, JEOL Ltd., Tokyo, Japan) using a secondary electron detector. 

## 3. Results and Discussion

### 3.1. Microstructures

**Figure 3:**
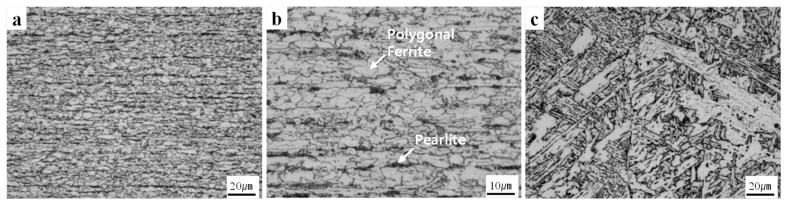
Microstructures of (**a**) base material, (**b**) high-magnification image of base material, and microstructures of (**c**) weld metal.

The microstructures of the base metal and weld metal are shown in Figure 3. The base metal had a rolled fine polygonal ferrite (PF)-discontinuous pearlite band (P) microstructure with the average grain size of 4.75 µm in diameter (Figure 3a,b), which are typical microstructures in low-carbon steels, and a tensile strength of 600 MPa. The weld metal showed coarse granular bainite with the average grain size of 33.8 µm in diameter (Figure 3c), but the structure was finer than that produced by gas tungsten arc welding process, because plasma arc welding process provides focused heat transfer that results in a lower heat input leading to improved penetration in the smaller heat-affected zone [32]. Previous studies observed fatigue fractures at the softened HAZ [5,6]; however, the inferior coarse WJ microstructures with low hardness produced using a welding consumable with low strength or a process with high heat input can cause fatigue fracture [3,4]. 

### 3.2. Mechanical Properties 

**Figure 4:**
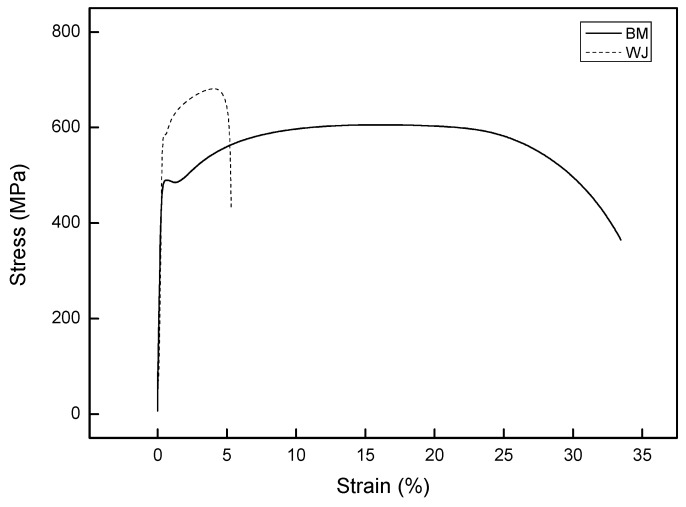
Tensile curves of base material (BM) and welded joint (WJ)specimens.

The tensile curves in Figure 4 show that the BM specimen had a yield strength and tensile strength up to 10% lower than those of the WJ specimen. However, the ductility of the BM specimen was 6.63 times that of the WJ specimen, as measured using Miranda’s equation [33], where the ductility was calculated from the ratio of the maximum displacement (*D_u_*) to the corresponding displacement at the onset of yielding (*D_y_*). At low strain ranges, materials of higher tensile strength generally have higher crack-growth resistance. By contrast, at high strain ranges, materials of higher ductility have higher crack-initiation resistance. Since strength level and ductility are usually inversely related, fatigue resistance involves a tradeoff among strength and ductility on the assumption of high fracture toughness without defects [34].

Previous studies showed that the fatigue properties are strongly associated with the mechanical properties of the material [35,36] and suggested that HCF resistance requires a high monotonic tensile strength [35], where the stress-based Basquin law is often used to estimate the HCF life. In contrast, LCF resistance requires a high monotonic ductility [36] and the strain-based Coffin–Manson law is often used to predict the LCF life. However, ELCF resistance requires both tensile strength and ductility, although the relative proportions are still being debated [37,38,39]. In early studies [15,40], the ELCF mode was characterized as a rapid reduction and exhaustion of the residual ductility at very large strain amplitudes, resulting in a short fatigue life (<100 cycles). As a result of this approach of including the ductility when considering fatigue failure in the ELCF mode, it is important to elucidate the fatigue resistance of the WJ specimen in order to determine whether a tolerable level of strain is exceeded during practical operation. 

The cyclic stress response behavior is determined by the mechanical properties of a material and should be considered when estimating the fatigue life. Comprehensive studies [41,42] have been conducted to identify the major parameters affecting fatigue life. First, it is necessary to separate cyclic strains into elastic and plastic components to elucidate their strain–stress behaviors under cyclic loading. The total strain amplitude (*Δε_t_*) is defined by Equation (1), where *Δε_p_* is the plastic strain amplitude, *Δε_e_* is the elastic strain amplitude, *E* is the elastic modulus (Young modulus), and *Δσ* is the stress amplitude.

(1) $$\frac{\Delta \varepsilon_{t}}{2}=\frac{\Delta \varepsilon_{p}}{2}+\frac{\Delta \varepsilon_{e}}{2}=\frac{\Delta \varepsilon_{p}}{2}+\frac{\Delta \sigma }{2E}$$

In addition, the elastic strain amplitude can be obtained from extracting the elastic strain components from the total strain amplitudes, as shown in Equation (2), where *σ′_f_* is the fatigue strength coefficient, *2N_f_* is the number of reversals to failure, and *b* is the fatigue strength exponent.

(2) $$\frac{\Delta \varepsilon_{e}E}{2}=\;{\sigma^{\prime }}_{f}{\left(2N_{f}\right)}^{b}$$

This equation is similar to Basquin’s equation [43] and has been shown to fit materials exceeding the fatigue limit well. It can be deduced that high *σ′_f_* and low *b* values increase the fatigue strength. The plastic strain components are described using the Coffin–Manson equation [44], as shown in Equation (3), where, *ε′_f_* is the fatigue ductility coefficient, and *c* is the fatigue ductility exponent.

(3) $$\frac{\Delta \varepsilon_{p}}{2}\;=\;{\varepsilon^{\prime }}_{f}{\left(2N_{f}\right)}^{c}$$

It is also clear that high *ε′_f_* and low *c* values lead to an increase in the fatigue life. Manson et al. [45] estimated the fatigue resistance by superposition of plastic and elastic components to give the fatigue strength at a specific strain amplitude. Thus, Equations (1)–(3) can be combined to obtain an expression between the total strain amplitude (*Δε_t_*), plastic strain amplitude (*Δε_p_*) and elastic strain amplitude (*Δε_e_*) as a function of the number of reversals to failure (*2N_f_*).

(4) $$\frac{\Delta \varepsilon_{t}}{2}=\frac{\Delta \varepsilon_{p}}{2}+\;\frac{\Delta \varepsilon_{e}}{2}={\sigma^{\prime }}_{f}{\left(2N_{f}\right)}^{c}+\;\frac{{\sigma \prime }_{f}}{E}{\left(2N_{f}\right)}^{b}$$

In addition, the hardening coefficient (*K′*) and cyclic hardening exponent (*n′*) are described by Ramberg–Osgood equation:

(5) $$\frac{\Delta \varepsilon_{t}}{2}=\frac{\Delta \varepsilon_{p}}{2}+\;\frac{\Delta \varepsilon_{e}}{2}={\left(\frac{\Delta \sigma }{2K^{\prime }}\right)}^{1/n^{\prime }}+\;\frac{\Delta \sigma }{2E}$$

The following equations for the material parameters can be derived from Equations (4) and (5).

(6) $$n^{\prime }=\frac{b}{c}$$

(7) $$K^{\prime }=\frac{\sigma_{f}^{\prime }}{{\left(\varepsilon_{f}^{\prime }\right)}^{n^{\prime }}}$$

**Figure 5:**
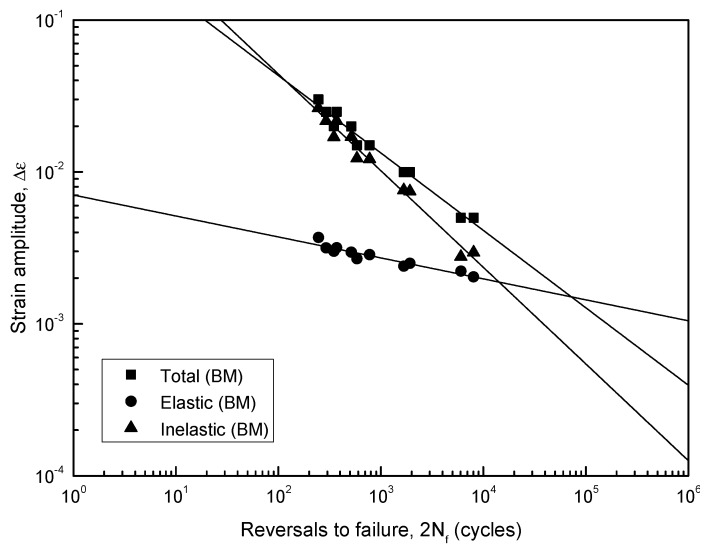
Fatigue strain-life curve obtained by superposition of elastic and plastic strains.

A linear Coffin–Manson relationship for the BM specimen was apparent, as plotted in Figure 5 on a log–log scale. The material parameters for the fatigue resistance (e.g., *σ′_f_*, *b, ε′_f_*, *c, n′* and *K′*) were obtained; *ε′_f_* and c from the slope and exponent of the plastic component of Equation (4), *σ′_f_* and low *b* from the slope and exponent of the elastic component of Equation (4), *n′* from Equation (6), and *K′* from Equation (7) were subsequently obtained, and listed in Table 2. 

Typically, the slopes of the plastic strain amplitudes of the BM specimen showed a drastic decrease as compared to those of the elastic strain amplitudes.

### 3.3. Fatigue Resistance at Welded Joint

**Figure 6:**
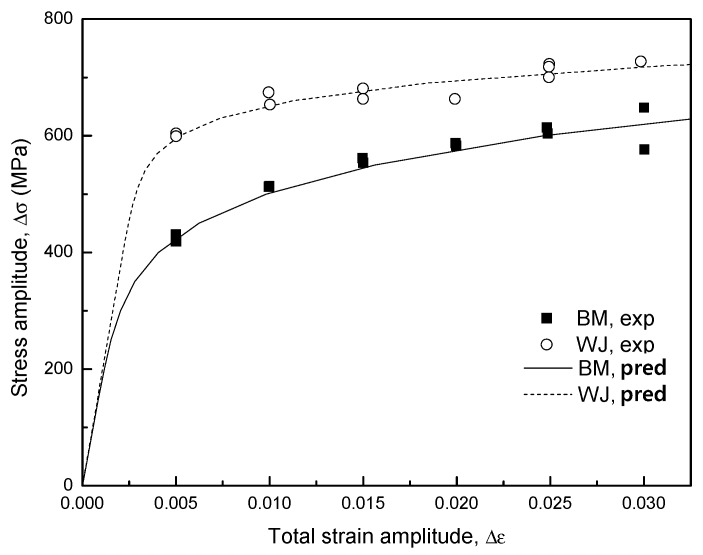
Cyclic stress–strain curves in the BM and WJ specimens.

The relationship of stable cyclic stress–strain for origin state material can be described by Equation (5) and the cyclic stress–strain response curves of the BM and WJ specimens are shown in Figure 6. Since the local stress–strain approach was developed for low-cycle fatigue, the prediction curves of fatigue lives in a life region share good agreement with experimental results. On the one hand, the stress values for the WJ specimen at all strain amplitudes were commonly higher than those for the BM specimen. We propose that different plastic deformation occurred in the WJ specimen because the WJ specimen has inhomogeneous complex microstructure or strength mismatching and its strain ranges should be modified to compare with the BM specimen.

Earlier studies have shown that the fatigue strength of welded joints is different from that in homogeneous base materials due to the welding defects [46] or weld toe [47], and the high level of residual stresses [48], complex microstructure, or strength mismatching [49,50].

It should be noted that the existence of welding defects such as porosities, lack of fusion, or lack of penetration may introduce cracks or crack-like defects, which were found to be areas preferable for fatigue crack initiation. In addition, the weld toe acts as the geometric stress concentrations and fatigue cracks primarily appear throughout the weld toe. In the present study, ultrasonic non-destructive testing was conducted for the welds to eliminate the welding defects that can cause the test results to become corrupted. In addition, the weld toe was removed by milling the surface of the welding seam to eliminate the stress concentration caused by geometric discontinuity. 

Residual stresses introduced by the welding process are detrimental and may reduce the fatigue strength by accelerating fatigue crack initiation and growth. In addition, the severe non-uniform temperature distribution in the HAZ around the weld can cause residual thermal and mechanical stresses that greatly influence the microstructural evolution in this region [1,2]. In other studies [51,52], however, eliminating residual stresses did not lead to an improvement in the low-cycle fatigue strength and its effect is not too critical and can be ignored as significant plasticity can occur from external loading that relaxes the residual stresses. In the present study, a SRHT was conducted at 600 °C for 15 min to remove even the minor effects of residual stresses.

For the smooth welded joint, mechanical inhomogeneity (complex microstructure or strength mismatching) can be the main reason that leads to the decrease of the low-cycle fatigue strength, although the stress concentrations and residual stresses were eliminated. In this test, at all the strain amplitudes, fatigue crack initiation and propagation occurred at the heat-affected zones, where the plasticity and ductility were weak. Although experimental tests were used to obtain material mechanical properties, it is difficult to clarify these parameters owing to size limitation of the weld zone. Because hardness is easier than other mechanical properties to determine and it does not require much space or material, many studies [53,54,55,56] used hardness distribution to determine the welded joint material properties as proportional to the base material properties, and have found a simple linear relation between the yield strength and hardness, including the following proportional form:

(8) $$K_{i}^{\prime }=K_{i}^{\prime }\frac{Hv_{i}}{Hv_{BM}},\;i=1,2,,,n$$

where *K′_i_*, *K′_BM_*, *Hv_i_* and *Hv_BM_* are the cyclic strength coefficient in the ith zone, the cyclic strength coefficient in the base material, the Vickers hardness in the ith zone, and the Vickers hardness value in the base material, respectively.

**Figure 7:**
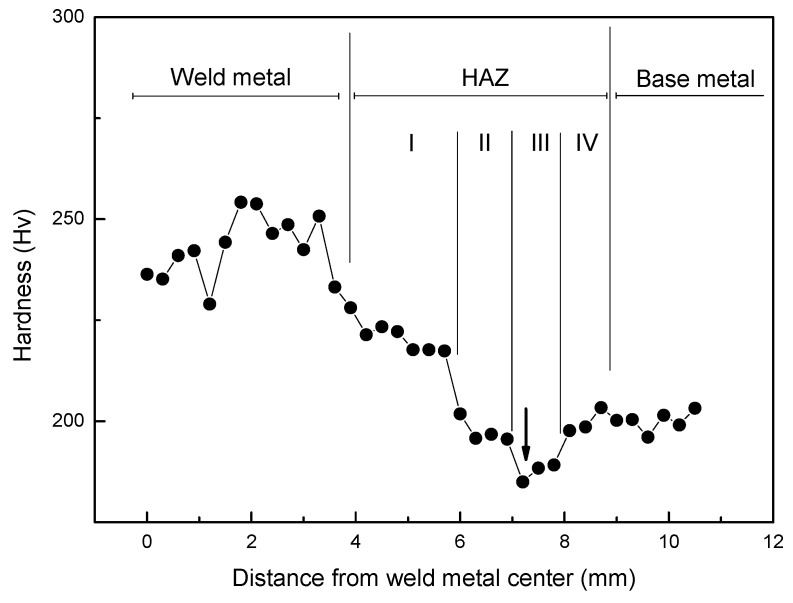
Microhardness profile of WJ specimen; arrow indicates fatigue fracture spot.

**Figure 8:**
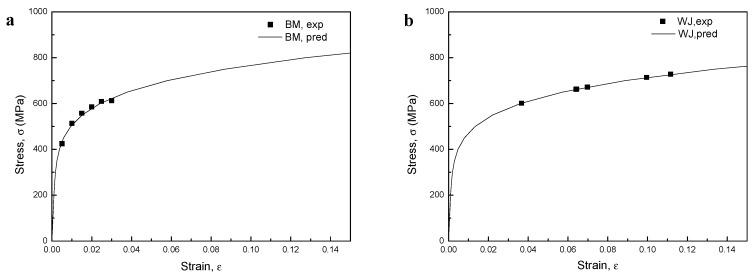
Cyclic stress–strain curves in the (**a**) BM and (**b**) WJ specimens.

**Figure 9:**
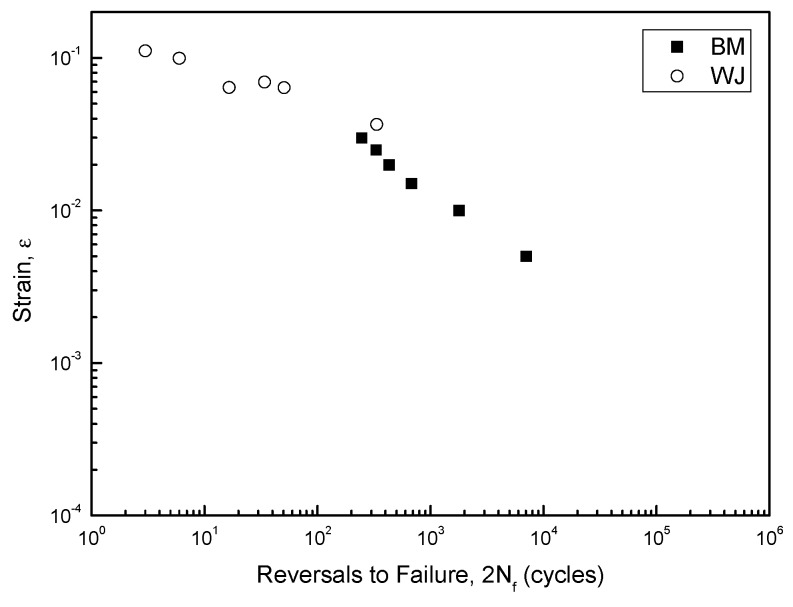
Fatigue strain-life curve obtained by superposition of BM and WJ specimens.

The fatigue parameters b, c of different zones are set as constant, and the fatigue strength coefficient *σ′_f_* is in proportion to hardness, that is

(9) $$\sigma_{fi}^{\prime }=\sigma_{fBM}^{\prime }\frac{Hv_{i}}{Hv_{BM}},\;i=1,2,,,n$$

where *σ′_f_*, *σ′_BM_* are the fatigue strength coefficient in the ith zone and the base material, respectively. For *K′*, it can be obtained by Equation (7). Here *ε′_f_* is treated as constant because *K′*, *σ′_f_* are in proportion to hardness value. The hardness distribution obtained for the welded joints is shown in Figure 7. In this study, it assumed all specimens have the same hardness distribution in the heat-affected zone and strain localization occurred. Hence there are total four HAZs near the weld metal, including Zone I, II, III, IV, and the Vickers hardness values decrease gradually and recover in this zone, from weld metal to base metal. The cyclic stress–strain parameters and strain-life curve parameters determined from hardness and base material parameters according to Equations (8) and (9) are shown in Table 3. The cyclic stress–strain curves and empirical data of the base material are shown in Figure 8a. The predicted fatigue lives in a life region share good agreement with experimental results. For the welded joint, the cyclic stress–strain curve at Zone III was used where the fatigue fracture occurred, and the strain range for experimental stress amplitude was adjusted as plotted in Figure 8b since the nominal strain cannot be directly matched to the local strain. After this rearrangement, fatigue strain-life curve obtained by superposition of the BM and WJ specimens was plotted in Figure 9 on a log–log scale. All WJ specimens were in higher strain ranges than the BM specimens.

### 3.4. Cyclic Stress Response

**Figure 10:**
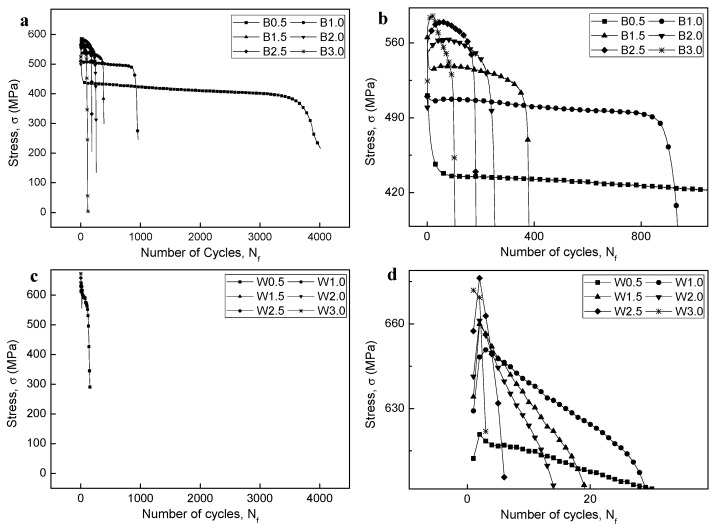
Cyclic stress response (CSR) curves of BM and WJ specimens; CSR curves of (**a**) BM, (**b**) high magnification of BM, (**c**) WJ, and (**d**) high magnification of WJ specimens.

Figure 10 shows the cyclic stress response curves of the BM and WJ specimens at different strain amplitudes. Different features were observed in the cyclic stress response curves of the BM and WJ specimens, although early fatigue failure commonly occurred with the increase in the strain amplitude. For example, initial cyclic softening was observed for the B0.5 sample, while initial cyclic hardening was observed for B1.0–3.0 (intermediate and large strains), as shown in Figure 10a,b. Note that for the BM specimen, initial cyclic hardening followed by cyclic stabilization was widely observed, although it became ambiguous at higher strains. In addition, a rapid drop in cyclic stress before fracture was observed, which was primarily due to the formation of macroscopic cracks and their unstable extension to fracture. In contrast, initial cyclic hardening was generally observed for specimens W0.5–2.5, showing cyclic hardening at high stress amplitudes with increasing strain, while no cyclic behavior was observed for W3.0 (Figure 10c,d). It should also be noted that, for specimens W0.5–2.5, initial cyclic hardening followed by cyclic softening readily occurred without cyclic stabilization, while there was no cyclic feature for W3.0.

**Figure 11:**
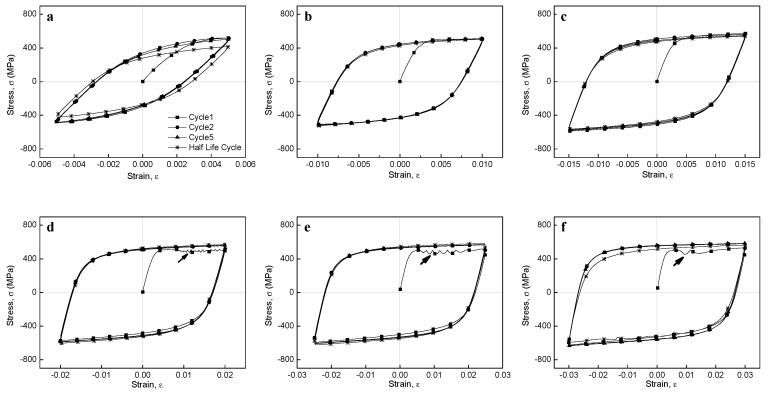
Hysteresis loops of the BM specimen at (**a**) 0.5%, (**b**) 1.0%, (**c**) 1.5%, (**d**) 2.0%, (**e**) 2.5%, and (**f**) 3.0% strain amplitudes; arrows indicate the serrations.

**Figure 12:**
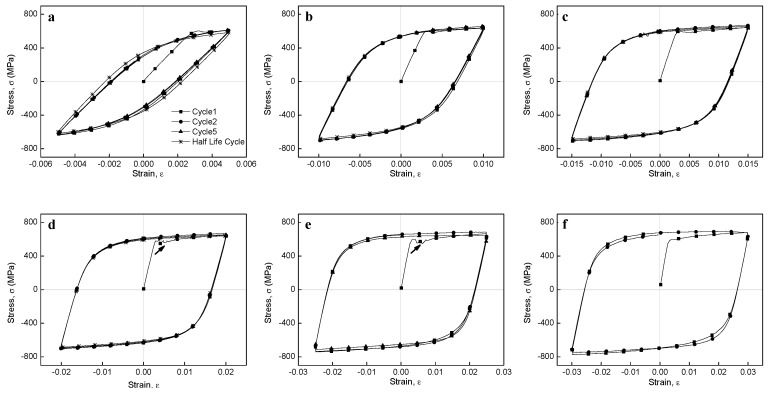
Hysteresis loops of the WJ specimen at (**a**) 0.5%, (**b**) 1.0%, (**c**) 1.5%, (**d**) 2.0%, (**e**) 2.5%, and (**f**) 3.0% strain amplitudes; arrows indicate the serrations.

The stress–strain hysteresis loops of the BM specimens at different strains are shown in Figure 11. It is important to note that the shape of the hysteresis loops changed from fusiform to semi-rectangular with increasing strain; hence, the fraction of the elastic component was higher than that of the plastic component. High strains resulted in large plastic strains that produced semi-rectangular hysteresis curves. In addition, at low cyclic load numbers, both maximum tensile and compressive stresses at the Y axis slightly increased with increasing cycle number (i.e., an apparent cyclic hardening phenomenon occurred), while at the half life cycles, maximum stresses decreased due to cyclic stabilization after initial cyclic hardening. In addition, the stress–strain hysteresis loops showed obvious serration flows at large strain amplitudes (B2.0 to B3.0), which was not observed for the lowest strain amplitudes (B0.5 to B1.0), although a rather attenuated serration flow was observed for B1.5. Periodic arrest and release of dislocations through dislocation pile-ups and dislocation cell wall structures have been suggested as the main triggers for serrated plastic flow [57], which will be discussed in detail later considering the TEM images (Section 3.9). The stress–strain hysteresis loops of the WJ specimen at different strains are shown in Figure 12. These curves showed a similar tendency to those of the BM specimens, changing from fusiform to semi-rectangular type with increasing strain amplitude. However, the stress levels at all strains in the WJ specimens were higher than those in the BM specimens due to the WJ specimens being exposed to a higher stress (as previously illustrated in Figure 8).

**Figure 13:**
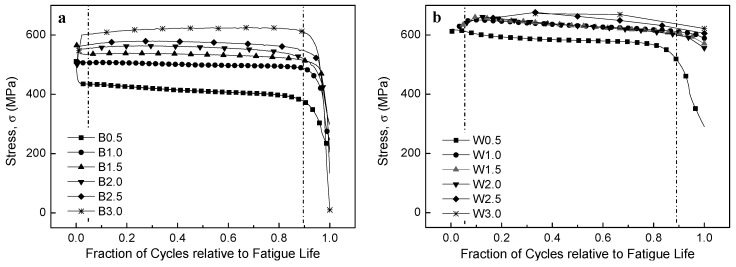
Stress amplitude as a function of fraction of cyclic number to fatigue life in (**a**) BM and (**b**) WJ specimens.

The stress amplitude as a function of fraction of cycles normalized to the fatigue life is depicted in Figure 13. The initial cyclic hardening or softening stage was relatively short, generally <5% of the total fatigue life. A rapid drop in the cyclic stress amplitude was observed for all curves just before fracture at the final cyclic stage, mostly within 10% of the fatigue life. It is possible that the fatigue lives were predominantly determined by the constant stress amplitudes in the intermediate stage, rather than at the stages of initial cyclic hardening (softening) or the final rapid drop.

### 3.5. Cyclic Hardening/Softening Behavior

**Figure 14:**
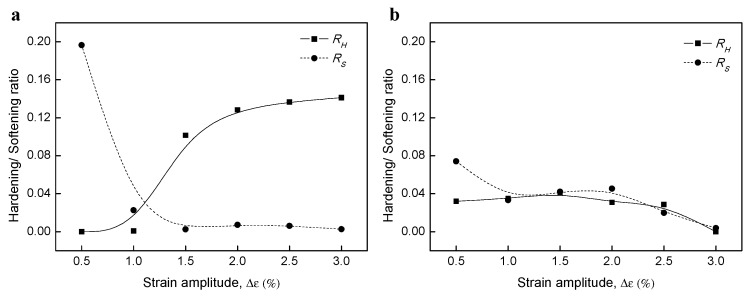
Hardening (*RH*) and softening (*RS*) ratio in (**a**) BM and (**b**) WJ specimens.

The ratios of cyclic hardening (*RH*) and cyclic softening (*RS*) can be expressed as follows [58]:

(10) $$RH\;=\left(\Delta \sigma_{max}-\Delta \sigma_{max}\right)/\Delta \sigma_{first}$$

(11) $$RS\;=\left(\Delta \sigma_{max}-\Delta \sigma_{half}\right)/\Delta \sigma_{max}\;$$

where, *Δσ_max_*, *Δσ_first_*, and *Δσ_half_* are the stress amplitudes at the maximum, first cycle, and half cycle, respectively, as shown in Figure 14. The BM specimens exhibited a high *RS* at low strain amplitudes (B0.5), while *RS* dramatically reduced, and *RH* increased with increasing strain amplitudes, where the ratios intersected at ±1.1% strain. The WJ specimens showed a high *RS* at low strain amplitudes (W0.5), while *RS* decreased to a similar level as *RH* at intermediate amplitudes (W1.0–2.5) and reached zero for W3.0. *RH* and *RS* are two important parameters in characterizing cyclic deformation behaviors of materials. That is, with high *RH* or *RS*, fatigue life can be longer by cyclic hardening or softening behaviors depending on materials or strain amplitudes. The difference between *RH* and *RS* and the relative fluctuation with strain amplitude for the WJ specimen were relatively small compared to those of the BM specimen; hence, we propose that the WJ specimen had lower hardening (softening) ability. 

**Figure 15:**
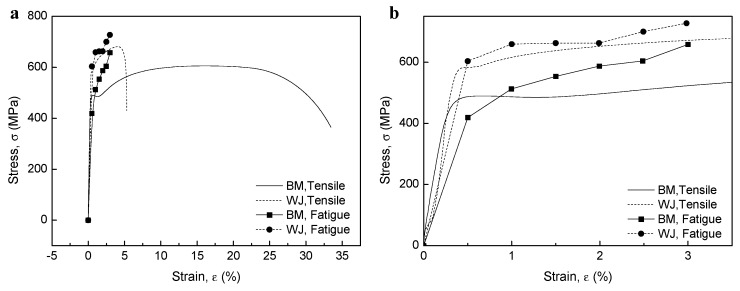
(**a**) Comparison of the cyclic stress–strain curve and the monotonic tensile stress–strain curves and (**b**) high magnification of 0 to 3.5% strain range.

Analyzing the differences between the cyclic strain and monotonic strain response is important as it can give information about the fatigue behavior of the material. Figure 15 compares the stress–strain curves of the cyclic stress and monotonic tension. For the BM specimens, the cyclic stress at small strains (B0.5) was slightly lower than the monotonic stresses at the same strain, while the values increased with increasing strain (B1.0–3.0). We propose that the CSR below the elastic strain showed cyclic stabilization subsequent to an initial cyclic softening as it was below the stress of monotonic tension. On the other hand, the CSR under plastic strain showed shorter cyclic stabilization after initial cyclic hardening with increasing strain as it was in the plastic deformation range. However, for the WJ specimen, cyclic stresses were slightly higher than monotonic stresses at all strains, resulting in initial strain hardening. 

### 3.6. Classification of Fatigue Fracture Mode

Classification of the fatigue fracture mode was based on the fatigue life and stress levels, modified from Figure 1. In this study, four modes were derived depending on the materials and strain amplitudes, i.e., HCF for B0.5, LCF for B1.0–3.0 and W0.5, ELCF for W1.0–2.5, and MF for W3.0. An overall scheme for determining the corresponding modes can be estimated from Figure 10 and Figure 15. The B0.5 specimen was under the elastic limit of the BM specimen (Figure 15b), which was in the stress-controlled HCF mode which showed initial cyclic softening followed by cyclic stabilization (Figure 10b). In addition, the B1.0–B3.0 and W0.5 specimens were over the elastic limit of the BM and WJ specimens, respectively, which were classified as strain-controlled LCF (cyclic hardening followed by cyclic stabilization or quasi-stabilization). However, the W0.5–2.5 specimens were over the elastic limit of the WJ specimens (Figure 15b) and were macroscopically in the stress concentration state represented by initial cyclic hardening, followed by cyclic softening and no stabilization (ELCF), which is evidence of ductility exhaustion. Sample W3.0 showed no cyclic damage behavior, but instead showed features similar to monotonic tensile conditions. This classification becomes more apparent when comparing the fracture features discussed in the next section.

### 3.7. Fracture Features of Base Material after Fatigue Tests 

Fatigue fracture features from fractography images are good indicators for identifying fracture modes. In the HCF mode, incipient cracks determine the fatigue life [19,20] and defining the transition between crack initiation and propagation is still controversial [59]. Meanwhile, the microscopic initial crack length and stress intensity at the crack tip are crucial for HCF resistance [21,60]. In addition, HCF cracks originate from a sub-surface inclusion with a diameter of several tens of micrometers [61], and a typical “fish-eye” fracture morphology is often observed. The strong residual compressive stress field has significant improvement in fatigue strength due to a lattice distortion, which causes the primary crack nucleation site (usually located at some defects such as a non-metallic inclusion) in HCF regions. The crack initiation begins by decohesion of the inclusion-matrix interface, and then the crack propagates forming a distinct fracture pattern known as the “fisheye”. A distinct crack propagation (fisheye) can be observed on the fracture surface. Approximately, the inclusion as crack nucleus in the HCF regime is located within fisheye. The existence of inclusion and fisheye is an essential feature of the interior failure in the HCF regime [62,63,64].

**Figure 16:**
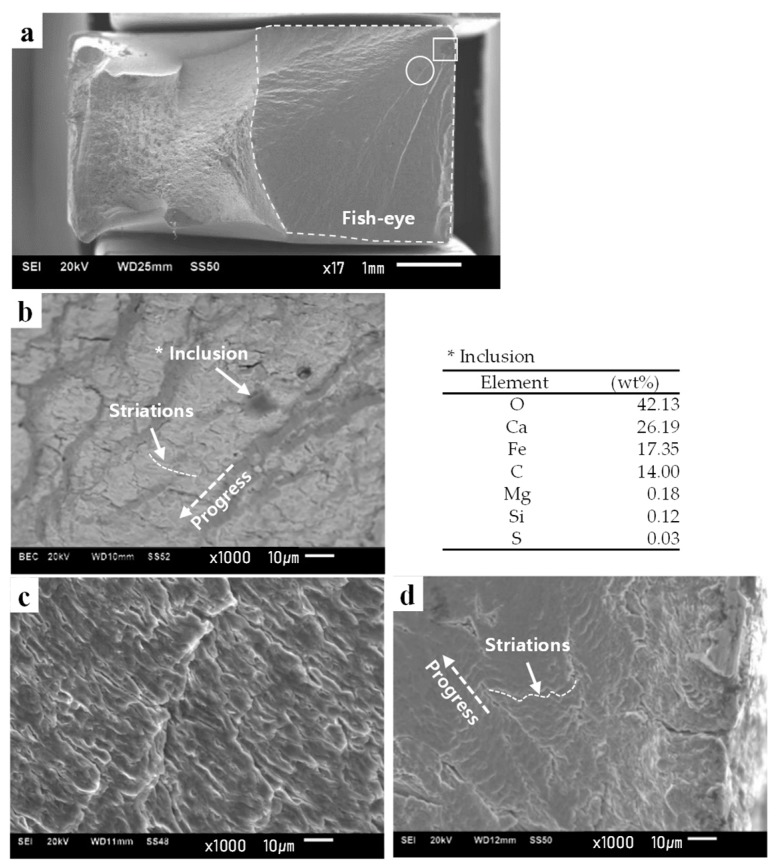
Fracture surfaces according to fracture modes; fracture surfaces in (**a**) low-magnification image of B0.5, the corresponding high-magnification image in area indicated by the (**b**) white box and (**c**) white circle in (**a**), (**d**) low-cycle fatigue (LCF) of B2.0 specimens * The loading direction is identically normal to the images.

Figure 16 shows representative images of fractured surfaces for each fracture mode observed in our experiments. In the HCF mode, a “fisheye” fracture morphology due to fatigue crack initiation at a sub-surface CaO composite with a diameter of 10 µm in the B0.5 specimen was observed (Figure 16a,b). Distinct fracture striations were observed at the fisheye fracture (Figure 16c). In addition, such inclusions originate from dephosphorization during steel making. In conventional steel making process, Iron ore raw materials contain impurities such as phosphorus and sulphur, which have been known to have detrimental impacts on the final steel properties. Accordingly, dephosphorization process is conducted by adding CaO/SiO_2_, which increases the slag basicity [65,66]. Thus, higher content of CaO accelerates the dephosphorization process in presence of FeO by dissolving the phosphates.

In the LCF mode, Forsyth et al. [67] differentiated two types of striations, Type A (ductile striations), which consist of light and dark bands, and Type B (brittle striations), which consist of river-like patterns with limited ductility. They also showed optical fractography images of the transition from ductile to brittle striations on a grain boundary facet of Al-5%Mg-4%Zn alloy [68]. In the present study, in the LCF mode of the BM specimen, striations with narrow spacings were conspicuous (Figure 16d), which may be the typical Type A striations.

**Figure 17:**
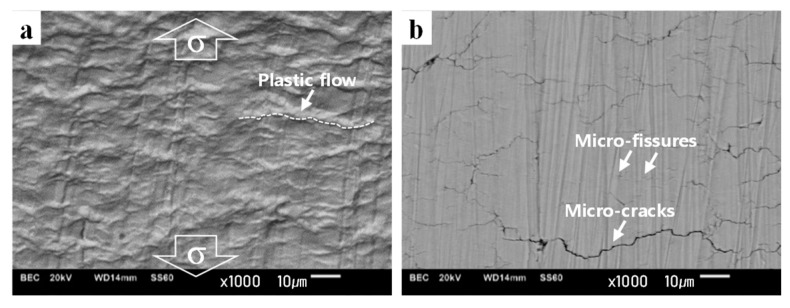
Surface damage morphology according to the fracture modes; surface damage morphologies in (**a**) high-cycle fatigue (HCF) of B0.5 and (**b**) low-cycle fatigue (LCF) of B2.0 specimens. * The loading direction is identically applied to the images.

To elucidate the fatigue damage features for each fracture mode, the surface damage of the specimens was observed after cyclic loading, as shown in Figure 17. In the HCF mode after cyclic loading tests, plastic flows induced by plastic deformations along the shear stress direction were developed (Figure 17a). In the LCF mode of the BM specimen, surface micro-fissures and cracks were observed, which implied that most surface grains of the BM specimen contained numerous intrusions and extrusions (Figure 17b); hence, a homogeneous deformation morphology was observed due to the uniform shear stress distribution during the fatigue test, which indicated that Stage I crack growth was the predominant fatigue crack propagation mechanism. 

### 3.8. Fracture Features of Welded Joint after Fatigue Tests 

In the LCF regime with increasing strain or in materials with low fatigue resistance, the striations became blurred and the spacings between them widened as a transition to the quasi-cleavage (QC) fracture mode occurred. On the other hand, at very large strains, or in materials with low LCF resistance [22,69], QC or monotonic fracture surfaces are often observed.

**Figure 18:**
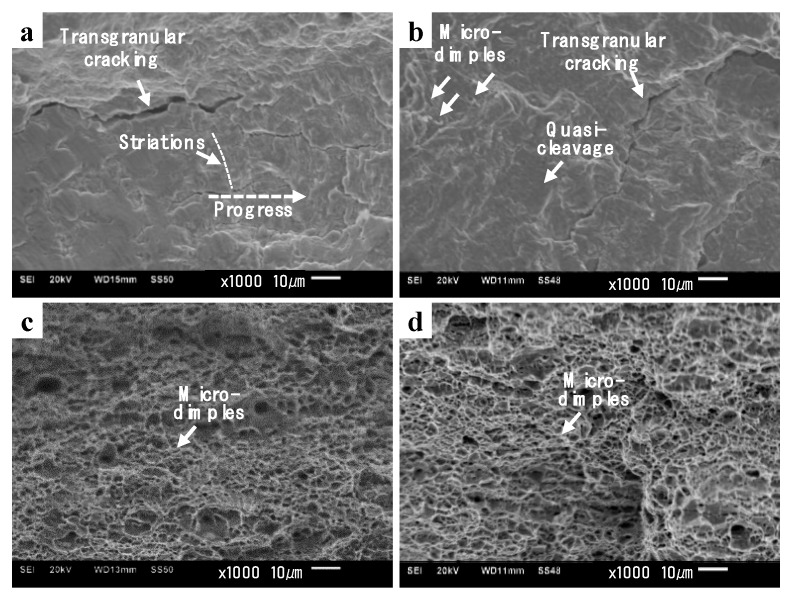
Fracture surfaces according to fracture modes; fracture surfaces in (**a**) low-cycle fatigue (LCF) of W0.5, (**b**) extremely low-cycle fatigue (ELCF) of W2.0, and (**c**) monotonic fracture (MF) of W3.0 specimen, and (**d**) MF in tensile test. * The loading direction is identically normal to the images.

In the LCF mode of the WJ specimen (W0.5), striations were blurred with primary transgranular cracks (Figure 18a), which may be the Type B striations. After initial cyclic hardening in the WJ specimen (Figure 10d), cyclic stabilization at constant strain amplitudes was observed (a balance between cyclic softening and dynamic recovery processes [70,71]), where cyclic strain localization occurred, resulting in frequent transgranular cracking [72,73]. In the ELCF mode of the WJ specimen (W2.0), a QC fracture with a few micro-dimples was observed (Figure 18b), which differed from the striations during the LCF test or MF during tensile tests. Cleavage fracture with micro-dimples has been attributed to ELCF at large cyclic strains, which usually leads to a short fatigue life of <100 cycles [15,74]. At large strains, the WJ specimen (W3.0) showed ductile fracture, characterized by micro-dimples (Figure 18c), which was identical to the fracture surface observed after tensile testing (Figure 18d). 

**Figure 19:**
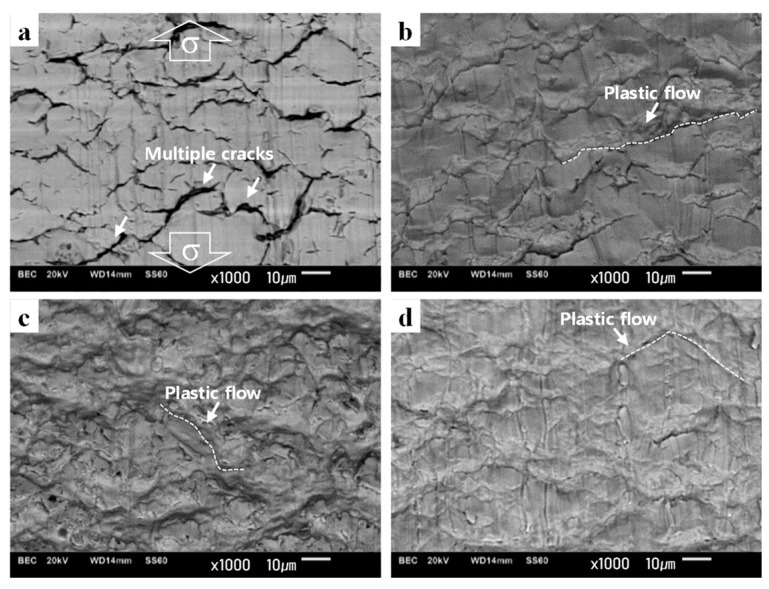
Surface damage morphology according to the fracture modes; surface damage morphologies in (**a**) low-cycle fatigue (LCF) of W0.5, (**b**) extremely low-cycle fatigue (ELCF) of W2.0, and (**c**) monotonic fracture (MF) of W3.0 specimens, and (**d**) MF in tensile test. * The loading direction is identically applied to the images.

The surface damage of the WJ specimens was also observed after cyclic loading and tensile tests, as shown in Figure 19. In the LCF mode of the WJ specimen, various well-developed cracks were clearly observed, where most cracks were connected at a higher angle of 38–69° (Figure 19a) than those in the LCF mode of the BM specimen (17–52°). Meanwhile, in the ELCF and MF modes of the WJ specimen, plastic flows induced by plastic deformations along the shear stress direction were developed (Figure 19b,c), which were identical to the surface morphology of the MF in the tensile test (Figure 19d); these were analogous to those in the HCF mode, but plastic flows at higher angles of 30–72° in the ELCF and MF modes are more likely to deform than those at lower angles of 11–30° in the HCF mode due to large applied cyclic tensile and compressive loads. 

Based on these observations, we concluded that the fatigue fracture and surface damage changed depending on the materials and strain amplitudes.

The ratio of crack initiation and crack propagation over the fatigue life depends on the failure mode. In the HCF mode, an incipient crack determined that the fatigue life and portions of crack initiation take up to 90% of fatigue life depending on the initiated crack size from an inclusion, material strength, and stress level [23,75].

In the case of the LCF mode, crack growth determines the fatigue life as the incipient cracks can be readily formed at large strain amplitudes [24]. The LCF phenomenon can be divided into four steps and two crack-growth stages in microstructural evolution [76]. Fatigue damage begins in Step 1, and symptoms of latent cracks appear, showing unstable cyclic hardening or softening. In Step 2, incipient cracks are formed on the planes of high shear stresses and gradually propagate inward; this is called “Stage I crack-growth.” In Step 3, cracks on the planes of high tensile stresses propagate dramatically perpendicular to the applied load; this is called “Stage II crack-growth.” In the final step, fracture occurs in the remaining reduction area after full crack growth. Micro-cracks nucleate and grow rapidly until they are arrested by grain boundaries; multiple cracks grow and interact until a fatal crack (above a critical length) occurs, at which point, all crack energies converge to form the fatal crack [77]. Generally, the strain amplitude and microstructural features are considered the key parameters determining the LCF fracture mode. The ratio of Stage I and Stage II crack growth is determined by the applied strain amplitude [78]. Stage I crack growth is dominant for smaller strains, which result in HCF behavior [79,80]. On the contrary, for larger strains, the fraction of Stage II crack growth increases, and the fracture behavior transitions to the ELCF mode. In addition, the MF mode was prevalent at sufficiently large strains, given that micro-dimples were solely observed after tensile tests.

### 3.9. Microstructural Evolution after Fatigue Tests

Persistent slip bands (PSBs) are observed within the HCF and LCF modes, which evolve via irreversible slips constituting intrusion and extrusion during cyclic tensile and compressive loads. PSBs are readily observed in face-centered cubic (FCC) materials as 12 close-packed slip systems running in {111} octahedral planes and the <110> direction. Various studies of PSBs have been conducted [81,82,83] owing to the ease of obtaining fractographic images and analyzing the dislocation behavior, especially in austenitic twinning-induced-plasticity and transformation-induced-plasticity steels [84,85]. However, body-centered cubic (BCC) materials are not close packed and the slip moves in the most atom-dense {110} planes, which have a lower atomic packing factor (APFα = 0.68) than that of FCC materials (APFγ = 0.74). Hence, a higher critical shear stress is required to initiate cracks in BCC structures than in FCC ones because in the initial stage, fatigue cracks initiate and propagate at the PSB–matrix interfaces under the shear stress in fracture Mode II, which acts parallel to the plane of the crack and perpendicular to the crack front [86,87]. Most studies concerning BCC materials were conducted using pure metals, as impurities introduced by alloying elements increase the complexity of the dislocation behavior. However, the findings of these studies are relevant as the presence of the small impurity concentrations does not completely alter the fundamental slip planes, but rather influences the level of cross-slips. Plastic deformation in BCC materials is controlled by the motion of screw dislocations in a periodic potential originating from their non-planar core structure. Studies of the slip systems of pure iron [88,89] reported the movement of screw dislocations on the {110} planes, inferring that edge dislocations can glide on {110} planes. Dislocation sources always move on {110} planes and require the motion of screw dislocations. In Fe–3%Si [90], slip probably occurred on the {110} plane, while other studies proposed {112} slip via edge dislocations [91], wavy {112} slip [92], or both {110} and {112} slip [93].

**Figure 20:**
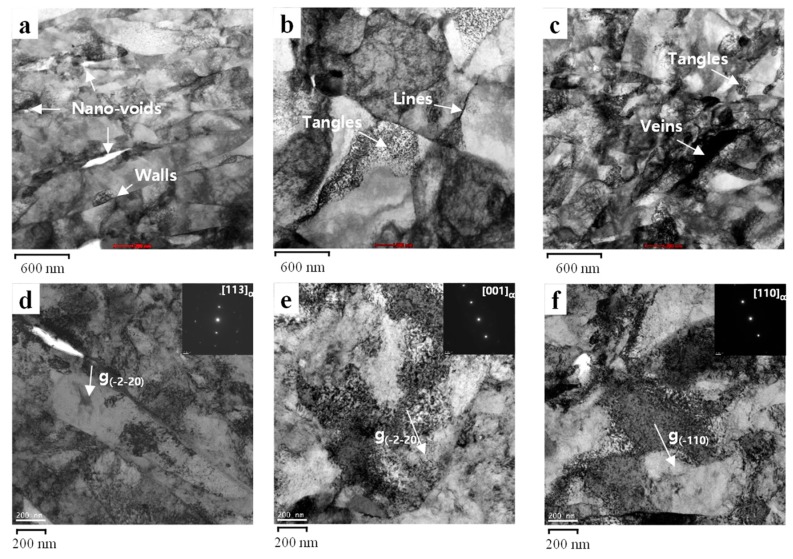
STEM images of (**a**) HCF, (**b**) LCF, and (**c**) ELCF (magnification of ×40,000), and (**d**–**f**) TEM images (magnification of ×13,500).

In the present study, in the base material, elongated subgrains and walls prevailed and some nano-voids at the dislocation lines or walls could be seen in the HCF mode (B0.5) (Figure 20a), and dislocation pile-ups at the dislocation lines with micro-voids were present on the {110} planes (Figure 20d). Owing to the largely elastic fatigue loadings in the HCF mode, it becomes easy to observe the energy dissipated by crack growth and microstructural change at the nanoscale. However, less dislocation tangles and veins were present in the LCF mode (B2.0), while incipient dislocation lines were observed (Figure 20b), and the diffraction patterns of the tangles in the LCF mode (Figure 18e). 

In the welded joint, heavy dislocation tangles and veins were observed along the strain-localized zone in the ELCF mode (W2.0) (Figure 20c), and they lie on the {110} planes (Figure 20f), where several dislocations and cross-slips can be easily activated during the deformation of the BCC material. High dislocation accumulation at the local zones such as dislocation tangles occurs in the elastic and plastic fatigue loadings.

### 3.10. Fatigue Features According to Fatigue Fractography and Surface Damage 

**Figure 21:**
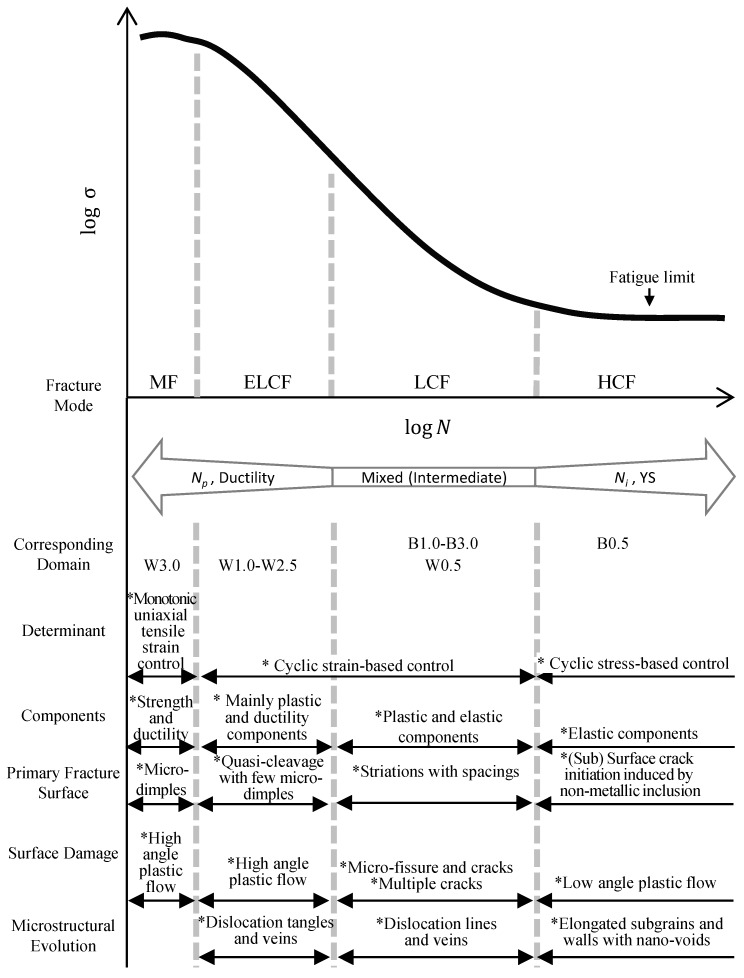
Fatigue properties according to classified fatigue modes.

The fatigue properties according to the classified fatigue fracture modes are illustrated in Figure 21. In the present study, the fatigue-fracture features such as fatigue fractography and surface damage depending on the fatigue fracture modes were observed on the BM and WJ specimens and classified into HCF for B0.5, LCF for B1.0–3.0 and W0.5, ELCF for W1.0–2.5, and MF for W3.0. An overall scheme for determining the corresponding modes can be estimated from the cyclic stress–strain curves and cyclic stress response (Figure 15). The B0.5 specimen was under the elastic limit of the BM specimen in the HCF mode. The B1.0–B3.0 and W0.5 specimens were over the elastic limit of the BM and WJ specimens, respectively, in the LCF mode. However, the W0.5–2.5 specimens were over the elastic limit of the WJ specimens in the ELCF mode. W3.0 showed no cyclic damage behavior, but instead showed features similar to monotonic tensile conditions.

Fractured surfaces for each fracture mode were observed in Figure 16. In the HCF mode, a CaO composite with a diameter of 10 µm was observed within a fisheye feature on the sub-surface of the B0.5 specimen (Figure 16a–c). In the LCF mode, striations with spacings were commonly observed (Figure 16d, Figure 18a). In the ELCF mode, a QC fracture with a few micro-dimples was observed (Figure 18b). At very large strains, it showed monotonic fracture, characterized only by micro-dimples (Figure 18c), which was identical to the fracture surface observed after static tensile testing (Figure 18d). 

The surface damages were also observed after cyclic loading and tensile tests. In the HCF mode, plastic flows induced by plastic deformations along the shear stress direction were developed (Figure 17a). In the LCF mode, micro-cracks were commonly observed (Figure 17b, Figure 19a). In the ELCF and MF modes, plastic flows induced by plastic deformations along the shear stress direction were developed (Figure 19b,c) at high angles ranging between 30–72°, which were identical to that in the static tensile test (Figure 19d). 

For microstructural evolution, in the HCF mode, elongated subgrains and walls prevailed and some nano-voids at the dislocation lines or walls could be seen (Figure 20a). However, in the LCF mode, less dislocation tangles and veins could be seen, while incipient dislocation lines were observed (Figure 20b). In addition, in the ELCF mode, heavy dislocation tangles and veins were observed along the strain-localized zone (Figure 20c).

In the HCF mode, dislocation pile-ups at the dislocation lines with micro-voids were present on the {110} planes (Figure 20d). The diffraction patterns of the tangles in the LCF mode (Figure 20e) and the dislocation veins in the ELCF mode (Figure 20f) show that they lie on the {110} planes, where several dislocations and cross-slips can be easily activated during the deformation of the BCC material.

## 4. Conclusions

Various strain amplitudes (*Δ*ε/2 = ± 0.5–3%) were imposed on BM and WJ specimens of a low-carbon steel with F+P microstructures to observe the transition of the fatigue fracture mode. The conclusions drawn from this study are as follows: 

(i) In the base material, the HCF and LCF fracture modes were observed through cyclic stress responses and fractography. The cyclic stress response in the HCF mode showed initial cyclic softening, followed by cyclic stabilization. The fractography showed inclusion-induced crack initiation at fish-eyes. In the microstructural evolution, elongated subgrains and walls prevailed and some nano-voids at the dislocation lines or walls could be seen.

(ii) The LCF fracture mode commonly was observed. The cyclic stress response showed initial cyclic hardening followed by cyclic stabilization, where fractography images showed obvious striations in the base material, while striations become blurred with transgranular cracking. In the microstructural evolution, dislocation lines and tangles could be seen.

(iii) In the welded joint, the ELCF mode at large strains showed no cyclic stabilization after initial cyclic hardening, which was characterized by QC fractures with a few micro-dimples and transgranular cracking. In the microstructural evolution, dislocation veins as well as tangles could be seen.

(iv) In the welded joint, the MF mode at large strain (±3%) predominantly showed micro-dimples (identical features to those observed after monotonic tensile loading). 

Fractography, surface damage morphologies, and microstructural evolution showed evidence of the transition of the fracture modes from LCF to ELCF and MF modes. These findings deepen the understanding of fatigue fracture behavior of low-carbon steels, and are expected to contribute to the use of this material in structures with improved durability and safety. For instance, structures might consider the damage tolerance design, carrying the localized loadings imposed by welding in addition to loads of its own axial stresses, and specifying the transition of the fatigue fracture mode depending on cyclic responses and fatigue fractography including the strain and fatigue life.

## Figures and Tables

**Table 1:** Chemical composition of coiled tubing (CT) steel (wt.%).

C	Mn	Si	P	S	Other Alloying Elements
0.12	0.87	0.36	0.01	0.001	Cr, Ni, Mo, Nb, Ti

**Table 2:** Fatigue parameters from equations.

Material	$\sigma_{f}^{\prime }$	b	$\varepsilon_{f}^{\prime }$	c	$n^{\prime }$	$K^{\prime }$
BM	1098	−0.1050	0.8232	−0.6360	0.1633	1125.6

**Table 3:** Cyclic stress–stain and fatigue parameters with hardness distribution.

Zone	Hardness(Hv)	E	$\sigma_{f}^{\prime }$	b	c	$K^{\prime }$	$n^{\prime }$
Base material	202	203.0	1098.0	−0.1050	−0.6360	1125.6	0.1633
Weld metal	243	203.0	1322.3	−0.1050	−0.6360	1355.6	0.1633
HAZ-I	221	203.0	1203.8	−0.1050	−0.6360	1234.0	0.1633
HAZ-II	198	203.0	1075.1	−0.1050	−0.6360	1102.2	0.1633
HAZ-III	188	203.0	1020.9	−0.1050	−0.6360	1046.5	0.1633
HAZ-IV	200	203.0	1088.2	−0.1050	−0.6360	1115.6	0.1633
